# Supplementary material for: New Variants of Squash Mosaic Viruses Detected in Human Fecal Samples
Source: Microorganisms. 2021 Jun 22;9(7):1349. doi: 10.3390/microorganisms9071349 (PMC8307838; doi:10.3390/microorganisms9071349)
Supplement: Supplementary file 1 [file microorganisms-09-01349-s001.zip › microorganisms-1226188-supplementary.pdf]

microorganisms

*Supplementary material:*

# New variants of Squash mosaic viruses detected in human fecal samples

**Fabiola Villanova [1,†], Roberta Marcatti [2,†], Mayara Bertanhe [2,3,†], Vanessa dos Santos Morais [2], Flavio Augusto de Padua Milagres [4,5], Rafael Brustulin [4,5], Emerson Luiz Lima Araújo [6], Roozbeh Tahmasebi [2], Steven S. Witkin [2,7], Xutao Deng [8,9], Eric Delwart [8,9], Ester Cerdeira Sabino [2], Cassio Hamilton Abreu-Junior [10], Élcio Leal [1,‡] and Antonio Charlys da Costa [2,‡]** †These authors contributed equally to this work. ‡These authors jointly supervised this work

1 Laboratório de Diversidade Viral, Instituto de Ciências Biológicas, Universidade Federal do Pará, Belem, PA 66075-000, Brazil; fvillanova@gmail.com; elcioleal@gmail.com (É.L.)
2 Departamento de Moléstias Infecciosas e Parasitárias and Instituto de Medicina Tropical da Faculdade de Medicina da Universidade de São Paulo, São Paulo, SP 05403-000, Brazil; robertamarcatti@gmail.com (R.M.); va.morais@usp.br (V.d.S.M.); roozbeh@usp.br (R.T.); switkin@med.cornell.edu (S.S.W.); sabinoec@gmail.com (E.C.S.); charlysbr@yahoo.com.br (A.C.d.C.)
3 School of Veterinary Medicine and Animal Science, University of Sao Paulo, São Paulo, SP 05508-270, Brazil; mayarabertanhe@globo.com
4 Instituto de Ciências Biológicas, Universidade Federal do Tocantins, Palmas, TO 77001-090, Brazil; flaviomilagres@uft.edu.br (F.A.d.P.M.); eu3rafael@gmail.com (R.B.)
5 Public Health Laboratory of Tocantins State (LACEN/TO), Palmas, TO 77016-330, Brazil
6 General Coordination of Public Health, Laboratories of the Strategic Articulation, Department of the Health, Surveillance Secretariat, Ministry of Health (CGLAB/DAEVS/SVS-MS), Brasília, DF 70719-040, Brazil; emerson.araujo@saude.gov.br
7 Department of Obstetrics and Gynecology, Weill Cornell Medicine, 1300 York Avenue, New York, NY 10065, USA
8 Vitalant Research Institute, 270 Masonic Avenue, San Francisco, CA 94143, USA; xdeng@vitalant.org (X.D.); Edelwart@Vitalant.org (E.D.)
9 Department Laboratory Medicine, University of California San Francisco, San Francisco, CA 94143, USA
10 Center of Nuclear Energy in Agriculture, Universidade de São Paulo, Piracicaba, SP 3400-970, Brazil; cahabreu@cena.usp.br
* Correspondence: elcioleal@gmail.com (E.L.)

**Keywords:** Squash mosaic virus, plant viruses, next generation sequencing, virome, public health

**Methodology:**

Sample Screening:
The samples described in this study were initially sent to the Public Health Laboratory of Tocantins (LACEN-TO), accompanied by a record of epidemiological findings such as demographic data (age, sex, date of collection) and clinical data (signs and symptoms) of the participants. The samples were stored at -20°C and the frozen fecal specimens were then taken to USP's Institute of Tropical Medicine (IMT/USP) to identify common enteric viruses as well as rare or potential new viruses through Next Generation Sequencing (NGS) investigation. The fecal specimens were screened for enteric pathogens (i.e., Rotavirus, Norovirus, Adenovirus, Astrovirus and Sapovirus), bacteria (i.e., *Escherichia coli* and *Salmonella* sp.), endoparasites (i.e., *Giardia* sp., *Taenia solium*), and helminths, using conventional culture techniques and commercial enzyme immunoassays, such as RotaScreenII® and AdenoScreen® EIA (Microgen Bioproducts Ltd, Watchmoor Point, Watchmoor Rd, Camberley GU15 3AD, UK). The identification of enteric pathogens such as bacteria and parasites was carried out by means of culture techniques and conventional parasitological tests such as Hoffman's method and fresh direct examination. To identify possible undetected enteric viruses, NGS techniques were applied to all samples using the method described below. Rotaviruses (n = 112), adenoviruses (n = 44), norovirus (n = 39), astroviruses (n = 8), and sapovirus (n = 8) were identified in some of these subjects.

Metagenomic preparation:
Initially, 50 mg of the human fecal sample was diluted in 500 μL of Hank's buffered saline solution (HBSS) and added to a 2 mL impact resistant tube containing C lysing matrix (MP Biomedicals, Santa Ana, CA, USA) and homogenized in a FastPrep-24 5G homogenizer (MP biomedicals, USA). The homogenized sample was centrifuged at 12,000×g for 10 min and approximately 300 μL of the supernatant was percolated through a 0.45 μm filter (Merck Millipore, Billerica, MA, USA) to remove bacterial and eukaryotic cells. Approximately 100 μL, PEG-it Virus Precipitation solution (System Biosciences, Palo Alto, CA, USA) was added to the filtrate and the content of the tube was gently homogenized and then incubated at 4°C for 24h. After the incubation period, the mixture was centrifuged at 10,000×g for 30 min at 4°C and the supernatant (~350 μL) was discarded. The granulate, rich in viral particles, was treated with a combination of nuclease enzymes (TURBO DNase and RNase Cocktail Enzyme Mix-Thermo Fischer Scientific, Waltham, MA, USA; Baseline-ZERO DNase DNase-Epicenter, Madison, WI, USA; Benzonase-Darmstadt, Darmstadt, Germany and RQ1 DNase-Free DNase and RNase A Solution-Promega, Madison, WI, USA) to digest unprotected nucleic acids. The resulting mixture was incubated at 37°C for 2h and the viral nucleic acids were extracted using a viral DNA/RNA kit ZR & ZR-96 (Zymo Research, Irvine, CA, USA), according to the manufacturer's instructions.
The synthesis of cDNA was performed with an AMV reverse transcription reagent (Promega, Madison, WI, USA). A second strand cDNA synthesis was performed using a large DNA polymerase I fragment (Klenow; Promega). Subsequently, a Nextera XT Sample Preparation Kit (Illumina, San Diego, CA, USA) was used to build a DNA library, which was identified using double barcodes. The library was purified using the ProNex® size selective purification system (Promega, WI, USA). Following the ProNex® purification, the quantity of each sample was normalized to ensure an equal representation of the library with the combined samples using the ProNex® NGS Library Quant Kit (Promega, WI, USA). For size range, Pippin Prep (Sage Science, Inc.) was used to select a 300 bp tablet (range 200 to 400 bp), which excluded very short and long fragments from the library. Before the generation of the cluster, the libraries were quantified again by qPCR using the ProNex® NGS Library Quant Kit (Promega, WI, USA). The library was sequenced in depth using a Hi-Seq 2500 sequencer (Illumina, CA, USA) with ends of 126 bp.

Metagenomic analysis and Contigs Assembly:
Bioinformatic analysis was performed according to the protocol previously validated [1,2]. Briefly, the non-viral sequences (i.e. human, bacterial, and fungal sequences) were removed using bowtie2. Later, the unmapped sequences were used for the reconstruction of viral genomes using an ensemble assembler, including SOAPdenovo2 (available at ftp://public.genomics.org.cn/BGI/SOAPdenovo2), Abyss (available at http://www.bcgsc.ca/platform/bioinfo/software/abyss.), meta-Velvet (available at http://metavelvet.dna.bio.keio.ac.jp/), CAP3 (available at http://www.mrc-lmb.cam.ac.uk/pubseq), Mira (https://sourceforge.net/projects/mira-assembler/files/MIRA/) and SPADES (http://cab.spbu.ru/software/spades/)

programs. The resulting singlets and contigs were analyzed using BLASTx to search for similarity to viral proteins in GenBank's Virus RefSeq. Also, the contigs were compared to the GenBank nonredundant nucleotide and protein database (BLASTn and BLASTx). The Sequences obtained with de novo assembly and identified in blast were then submitted mapping with the Geneious R9 Software (Biomatters Ltd L2, 18 Shortland Street Auckland, 1010, New Zealand), so that the generated sequence did not generate a biased or chimera sequence.

**Results:**

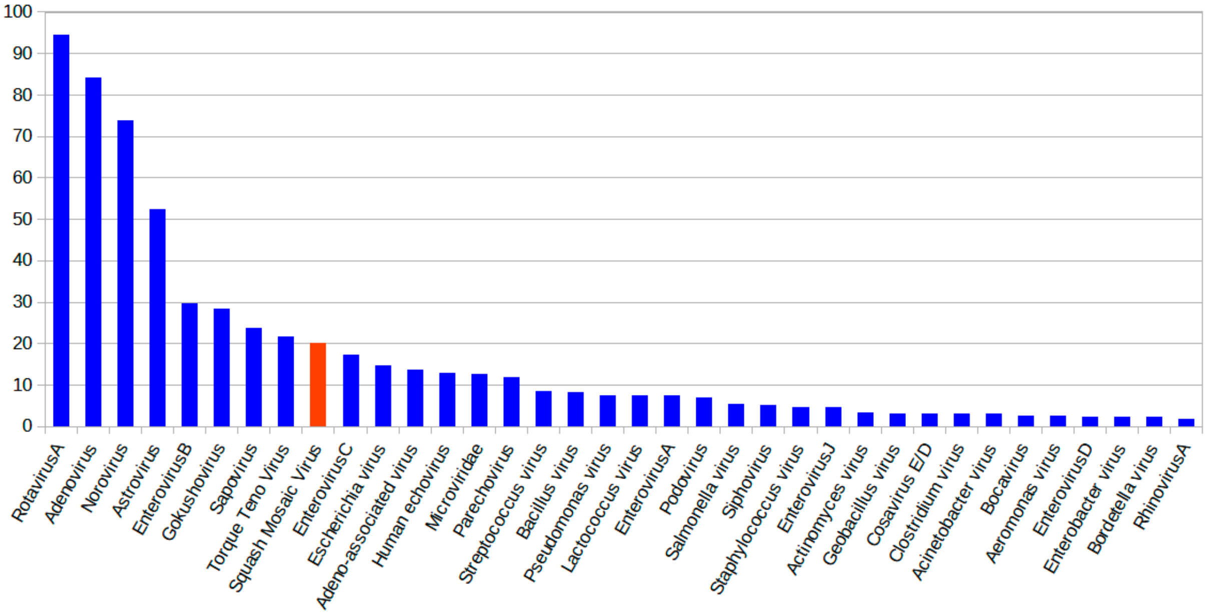


**Figure S1.** Viruses in human faecal samples. The y-axis show the percentages of viruses in all samples. Only the viral reads longer than 100bp and detected in more than 2% of samples are shown.

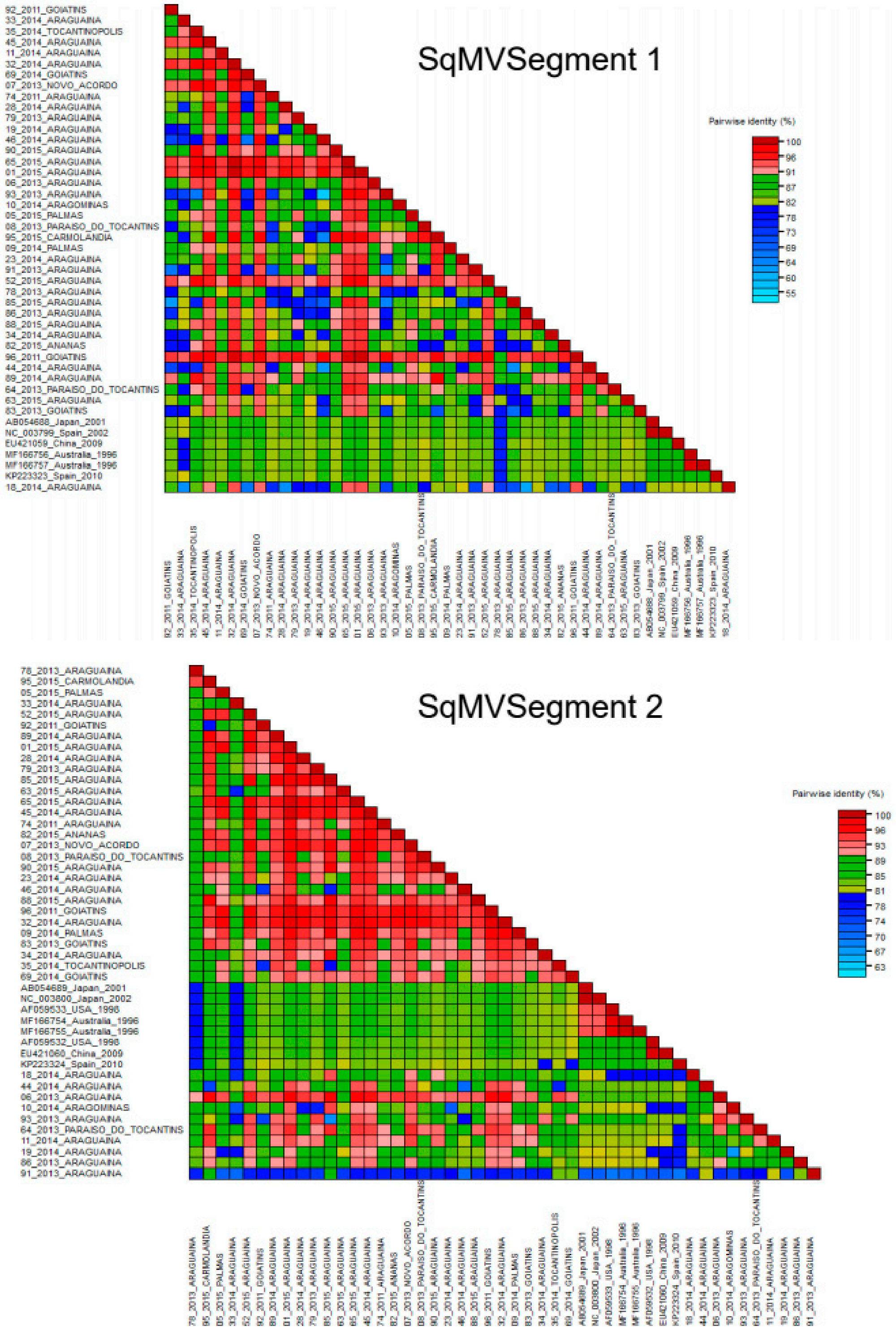


**Figure S2.** Similarity matrix of genomic segments of the Squash mosaic viruses (SqMV). Nucleotide similarity matrix of sequences of SqMV. The similarity of all pairs of sequences are indicated in colors according to the scale in the figure. To estimate the similarity of sequences we used a pair-wise method implemented in the program SDT [3]

**Table S1.** General characteristics of patients with Squash mosaic viruses

| Identification | Date of birth | Gender | Location | Symptoms | Other viruses identified by NGS* |
|---|---|---|---|---|---|
| TO-001 | 2015-03-26 | Male | Araguaina | Diarrhea | Rotavirus A, adenovirus and mamastrovirus |
| TO-005 | 2014-01-30 | Female | Porto Nacional | No data | Enterovirus B, gokushovirus, rotavirus A, enterovirus C, husavirus, norovirus, torque teno virus, aichivirus A and enterovirus J |
| TO-006 | 2012-05-19 | Female | Araguaina | Diarrhea and vomiting | Rotavirus A and adenovirus |
| TO-007 | 2012-10-19 | Male | Novo Acordo | No data | Rotavirus A, enterovirus B, adenovirus, gokushovirus, norovirus, torque teno virus, cosavirus A, astrovirus and podovirus |
| TO-008 | 2010-04-12 | Male | Paraíso do Tocantins | No data | Norovirus, human parechovirus, rotavirus A, sapovirus, adenovirus and astrovirus |
| TO-009 | 2013-08-22 | Male | Palmas | No data | Gokushovirus, rotavirus A, adenovirus, sapovirus, mamastrovirus, norovirus, myoviridae, siphovirus, siphovirus and enterovirus A |
| TO-010 | 2013-05-31 | Male | Aragominas | Diarrhea, vomiting and fever | Mamastrovirus |
| TO-011 | 2013-06-17 | Male | Araguaina | Diarrhea and fever | Rotavirus A and sapovirus |
| TO-018 | 2013-04-02 | Male | Araguaina | No data | Rotavirus A, enterovirus C, norovirus and adenovirus |
| TO-019 | 2011-01-14 | Male | Araguaína | Diarrhea, vomiting and fever | Rotavirus A, adenovirus, mamastrovirus and siphovirus |
| TO-023 | 2012-02-21 | Male | Araguaína | Diarrhea, vomiting and fever | Rotavirus A, enterovirus C, enterovirus B, norovirus, adenovirus, astrovirus, enterovirus D, potato virus M and cosavirus |

| | | | | | |
|---|---|---|---|---|---|
| TO-028 | 2014-04-23 | Male | Araguaína | Diarrhea | Rotavirus A, adenovirus, torque teno virus and norovirus |
| TO-032 | 2013-08-09 | Fema le | Araguaína | Diarrhea, vomiting and fever | Rotavirus A, adenovirus, enterovirus B, norovirus and mamastrovirus |
| TO-033 | 2011-03-06 | Fema le | Araguaína | Diarrhea, vomiting and fever | Norovirus, rotavirus A, enterovirus B, enterovirus C, adenovirus, human enterovirus 113, torque teno virus, astrovirus, enterovirus J and enterovirus A |
| TO-034 | 2012-11-18 | Male | Araguaína | Diarrhea, vomiting and fever | Rotavirus A, gokushovirus, podovirus, adenovirus, norovirus and halovirus |
| TO-035 | 2012-07-25 | Male | Tocantinópolis | Diarrhea | Rotavirus A |
| TO-044 | 2013-06-17 | Fema le | Araguaina | Diarrhea and vomiting | Adenovirus, rotavirus A and norovirus |
| TO-045 | 2011-10-28 | Fema le | Araguaína | Fever and vomiting | Rotavirus, adenovirus and norovirus |
| TO-046 | 2014-05-24 | Fema le | Araguaina | Diarrhea and vomiting | Rotavirus A, sapovirus, adenovirus, norovirus, astrovirus, gokushovirus, enterovirus C, enterovirus B and human parecho virus |
| TO-052 | 2010-11-17 | Fema le | Araguaína | Diarrhea, vomiting and fever | Rotavirus A, gokushovirus, adenovirus, norovirus, sapovirus and astrovirus |
| TO-063 | 2015-02-11 | Male | Araguaína | Diarrhea, vomiting and fever | Rotavirus A, adenovirus, norovirus and mamastrovirus |
| TO-064 | 2013-02-26 | Fema le | Paraíso do Tocantins | No data | Rotavirus A and adenovirus |
| TO-065 | 2014-11-24 | Male | Araguaína | Diarrhea and vomiting | Rotavirus A, adenovirus, mamastovirus, norovirus and enterovirus C |
| TO-069 | 2013-10-06 | Male | Goiatins | Diarrhea and fever | Adenovirus, rotavirus A, norovirus and mamastrovirus |
| TO-074 | 2010-06-16 | Male | Araguaína | No data | Adenovirus, rotavirus A and norovirus |

| | | | | | |
|---|---|---|---|---|---|
| TO-078 | 2012-09-21 | Male | Araguaina | Diarrhea, vomiting and fever | Adenovirus, rotavirus A, norovirus and mamastrovirus |
| TO-079 | 2012-11-30 | Male | Araguaina | Diarrhea and vomiting | Adenovirus, rotavirus A and enterovirus B |
| TO-082 | 2014-12-12 | Fema le | Ananás | No data | Rotavirus A and adenovirus |
| TO-083 | 2012-07-03 | Male | Barra do Ouro | Diarrhea, vomiting and fever | Sapovirus, norovirus, rotavirus A, human parechovirus, adenovirus, torque teno virus, astrovirus and enterovirus B |
| TO-085 | 2011-07-18 | Male | Araguaína | Diarrhea, vomiting and fever | Rotavirus A, adenovirus and norovirus |
| TO-086 | 2012-07-31 | Fema le | Araguaína | Diarrhea, vomiting and fever | Rotavirus A and adenovirus |
| TO-088 | 2010-09-08 | Fema le | Araguaína | Diarrhea, vomiting and fever | Norovirus, human bocavirus, enterovirus B, adenovirus, mamastrovirus and rotavirus A |
| TO-089 | 2010-04-12 | Male | Araguaína | Diarrhea and fever | Rotavirus A, adenovirus, norovirus and sapovirus |
| TO-090 | 2015-02-06 | Male | Araguaína | Diarrhea, vomiting and fever | Adenovirus, rotavirus A and enterovirus B |
| TO-091 | 2012-06-11 | Fema le | São Geraldo do Araguaia | Diarrhea and fever | Enterovirus B, rotavirus A, gokushovirus, torque teno virus, enterovirus C, adenovirus, norovirus, husavirus and astrovirus |
| TO-092 | 2005-10-04 | Male | Goiatins | No data | Rotavirus A, norovirus, adenovirus and sapovirus |
| TO-093 | 2011-05-21 | Fema le | Araguaína | Diarrhea, vomiting and fever | Rotavirus A, enterovirus C, sapovirus, adenovirus, gokushovirus, podovirus, picobirna virus and norovirus |
| TO-095 | 2011-10-30 | Fema le | Carmolândia | No data | Rotavirus A, adenovirus, human parechovirus, sapovirus, norovirus, salivirus A, astrovirus, gokusho virus and enterovirus B |

| | | | | | |
|---|---|---|---|---|---|
| TO-096 | 1990-08-01 | Male | Goiatins | No data | Adenovirus and rotavirus A |

The presence of bacteriophages was also identified, such as pseudomonas virus, escherichia virus and clostridium virus; however these are not included in the table, as they are not the focus of the study.

**Table S2.** Differences in the amino acid composition in the genomic segments of the SqMV

| Amino acid replacement* | |
|---|---|
| Genomic segment 1 | Genomic segment 2 |
| 5(N→H); 11(S→G); 29(T→A); 72(M→L); 75(H→F); 92(T→A); 167(E→D); 407(V→I); 481(T→S); 500(A/T→S); 648(F→Y); 657(N→D);658(A/V→T); 752(M→I); 800(T→A); 815(T/S→P);842(G/E→D); 889(S/G→C); 1114(V→I); 1139(H→N); 1261(D→N); 1270(E→D); 1318(A/T→V); 1326(Y→H); 1327(H→Q); 1331(I/T→V); 1334(E/Q→D); 1338(K→R); 1362(T→A); 1397(L→P); 1398(A→F); 1399(C→H); 1400(Q→T); 1453(G→I); 1454(G→A); 1456(Q→R); 1458(S→Q); 1506(E→R); 1507(I→K); 1508(L→S); 1509(V→R); 1580(P→S); 1588(S→D); 1725(I→V); 1756(H→N); 1828(P→S); 1835(T→A); 1506(E→R). | 7(Q→R); 60(I→V); 71(A→G); 110(A→T); 111(E→G); 128(K→Q); 276(P→S); 357(Q→K); 367(S→A); 414(S→C); 416(S→P); 420(L→S); 432(E→S); 486(T→A); 520(R→K); 529(I→V); 625(R→K); 781(L→V); 917(R→K); 923(N→S). |

*) These substitutions refer to the differences between worldwide SqMV and the Brazilian sequences.

Table S3. General features of SqMV sequences identified in this study.

| Sequence name | Sequence length / Coverage* | |
|---|---|---|
| | Segment 1 | Segment 2 |
| 01_2015_ARAGUAINA | 5873 / 330 | 3354 / 610 |
| 05_2015_PALMAS | 3879 / 283 | 2094 / 410 |
| 06_2013_ARAGUAINA | 4526 /610 | 2786 / 345 |
| 07_2013_NOVO ACORDO | 5818 / 980 | 3175 / 764 |
| 08_2013_PARAISO DO TOCANTINS | 2723 / 321 | 2154 / 244 |
| 09_2014_PALMAS | 4104 / 654 | 2930 / 731 |
| 10_2014_ARAGOMINAS | 3570 / 400 | 2371 / 342 |
| 11_2014_ARAGUAINA | 3715 / 167 | 1992 / 180 |
| 18_2014_ARAGUAINA | 2899 / 211 | 2433 / 230 |
| 19_2014_ARAGUAINA | 3015 / 252 | 2179 / 345 |
| 23_2014_ARAGUAINA | 3471 / 330 | 2072 / 550 |
| 28_2014_ARAGUAINA | 2878 / 180 | 2143 / 237 |
| 32_2014_ARAGUAINA | 5860 / 710 | 3355 / 620 |
| 33_2014_ARAGUAINA | 3198 / 215 | 1870 / 425 |
| 34_2014_ARAGUAINA | 3253 / 145 | 2594 / 217 |
| 35_2014_TOCANTINOPOLIS | 690 / 97 | 890 / 102 |

| | | |
|---|---|---|
| 44_2014_ARAGUAINA | 1186 / 222 | 838 / 199 |
| 45_2014_ARAGUAINA | 5877 / 546 | 3641 / 288 |
| 46_2014_ARAGUAINA | 1220 / 126 | 1144 / 134 |
| 52_2015_ARAGUAINA | 5673 / 677 | 2930 / 288 |
| 63_2015_ARAGUAINA | 2921 / 279 | 2246 / 178 |
| 64_2013_PARAISO DO TOCANTINS | 3607 / 165 | 2255 / 242 |
| 65_2015_ARAGUAINA | 5844 / 655 | 3354 / 346 |
| 69_2014_GOIATINS | 2654 / 398 | 1813 / 289 |
| 74_2011_ARAGUAINA | 2823 / 212 | 2152 / 301 |
| 78_2013_ARAGUAINA | 3975 / 314 | 3102 / 277 |
| 79_2013_ARAGUAINA | 3992 / 342 | 2426 / 267 |
| 82_2015_ANANAS | 2801 / 269 | 2500 / 192 |
| 83_2013_GOIATINS | 2843 / 267 | 2625 / 323 |
| 85_2015_ARAGUAINA | 924 / 79 | 533 / 93 |
| 86_2013_ARAGUAINA | 2404 / 162 | 1534 / 168 |
| 88_2015_ARAGUAINA | 3618 / 332 | 2768 / 431 |
| 89_2014_ARAGUAINA | 5580 / 546 | 3135 / 723 |
| 90_2015_ARAGUAINA | 5140 / 511 | 3065 / 542 |
| 91_2013_ARAGUAINA | 1572 / 324 | 1980 / 452 |
| 92_2011_GOIATINS | 1836 / 105 | 1401 / 204 |
| 93_2013_ARAGUAINA | 1336 / 145 | 585 / 77 |
| 95_2015_CARMOLANDIA | 418 / 86 | 457 / 108 |
| 96_2011_GOIATINS | 5941 / 453 | 3559 / 365 |

*) Mean coverage of the each assembly estimated with the Geneious R9 Software (Biomatters Ltd L2, 18 Shortland Street Auckland, 1010, New Zealand)

**References:**


1. Deng, X. *et al.* An ensemble strategy that significantly improves de novo assembly of microbial genomes from metagenomic next-generation sequencing data. *Nucleic Acids Res.* **43**, e46 (2015).

2. Altan, E. *et al.* Complex Virome in a Mesenteric Lymph Node from a Californian Sea Lion (Zalophus Californianus) with Polyserositis and Steatitis. *Viruses* **12**, 793 (2020).

3. Muhire, B.M.; Varsani, A.; Martin, D.P. SDT: a virus classification tool based on pairwise sequence alignment and identity calculation. PLoS One. 2014, 9(9), e108277, doi: 10.1371/journal.pone.0108277.
